# Lack of Association Between Toll-like Receptor 2 Polymorphisms (R753Q and A-16934T) and Atopic Dermatitis in Children from Thrace Region of Turkey

**DOI:** 10.4274/balkanmedj.2015.1253

**Published:** 2017-05-15

**Authors:** Ceren Can, Mehtap Yazıcıoğlu, Hakan Gürkan, Hilmi Tozkır, Adnan Görgülü, Necdet Hilmi Süt

**Affiliations:** 1 Department of Pediatric Allergy and Immunology, Trakya University School of Medicine, Edirne, Turkey; 2 Department of Genetics, Trakya University School of Medicine, Edirne, Turkey; 3 Department of Dermatology, Trakya University School of Medicine, Edirne, Turkey; 4 Department of Biostatistics, Trakya University School of Medicine, Edirne, Turkey

**Keywords:** Allergy, atopic dermatitis, polymorphism, toll-like receptor

## Abstract

**Background:**

Atopic dermatitis is the most common chronic inflammatory skin disease. A complex interaction of both genetic and environmental factors is thought to contribute to the disease.

**Aims:**

To evaluate whether single nucleotide polymorphisms in the TLR2 gene c.2258C>T (R753Q) (rs5743708) and TLR2 c.-148+1614T>A (A-16934T) (rs4696480) (NM_0032643) are associated with atopic dermatitis in Turkish children.

**Study Design:**

Case-control study.

**Methods:**

The study was conducted on 70 Turkish children with atopic dermatitis aged 0.5-18 years. The clinical severity of atopic dermatitis was evaluated by the severity scoring of atopic dermatitis index. Serum total IgE levels, specific IgE antibodies to inhalant and food allergens were measured in both atopic dermatitis patients and controls, skin prick tests were done on 70 children with atopic dermatitis. Genotyping for TLR2 (R753Q and A-16934T) single nucleotide polymorphisms was performed in both atopic dermatitis patients and controls.

**Results:**

*Cytosine-cytosine and cytosin-thymine* genotype frequencies of the TLR2 R753Q single nucleotide polymorphism in the atopic dermatitis group were determined as being 98.6% and 1.4%, cytosine allele frequency for TLR2 R753Q single nucleotide polymorphism was determined as 99.29% and the thymine allele frequency was 0.71%, thymine-thymine, thymine-adenine, and adenine-adenine genotype frequencies of the TLR2 A-16934T single nucleotide polymorphism were 24.3%, 44.3%, and 31.4%. The thymine allele frequency for the TLR2 A-16934T single nucleotide polymorphism in the atopic dermatitis group was 46.43%, and the adenine allele frequency was 53.57%, respectively. There was not statistically significant difference between the groups for all investigated polymorphisms (p>0.05). For all single nucleotide polymorphisms studied, allelic distribution was analogous among atopic dermatitis patients and controls, and no significant statistical difference was observed. No homozygous carriers of the TLR2 R753Q single nucleotide polymorphism were found in the atopic dermatitis and control groups.

**Conclusion:**

The TLR2 (R753Q and A-16934T) single nucleotide polymorphisms are not associated with atopic dermatitis in a group of Turkish patients.

Atopic dermatitis (AD) is the most widespread chronic inflammatory skin disease, and its rising frequency is an important health problem worldwide (1). It is assumed that an interplay of both genetic and environmental factors contributes to AD, but the cause of disease is still not known (2). The importance of genetic factors in AD (OMIM *603165) has been reported in studies of twins (3). Twin concordance studies have shown genetic factors to be the principal factor in the development of AD. Genetic contribution to the disease is predicted to be 80% (4). The concordance rate of AD is higher in monozygotic twins compared with dizygotic twins. Parental history is known to be an important risk factor for AD. If AD is present in one parent, the incidence of AD is twofold, and threefold in cases where both parents have AD (3).

In spite of these findings, a common defective pathway leading to the clinical phenotype of AD has not yet been defined. Genome-wide association studies have shown a number of genetic susceptibility loci. Many susceptible loci enriched within the AD population have been identified either within or near genes that are crucial to innate immunity, Th2-mediated inflammation, and the skin barrier function, which indicates the significance of these in AD pathogenesis (4).

Pattern recognition receptors can identify pathogen-associated molecular patterns (PAMPs) and stimulate the innate immune system (5). Toll-like receptors (TLRs) are among the host defence molecular regulators and can identify different PAMPs of microorganisms (6), like lipopolysaccharide, lipopeptide, RNA, and methylated CpG DNA; this in turn causes inflammatory responses that stimulate the flow of interleukins and other pro-inflammatory mediators (7).

TLRs, a kind of transmembrane protein (6), perform a mediating role between innate and adaptive immunity as they induce dendritic cells to mature and activate naïve cells, mainly swapping the adaptive immune response for Th1 polarisation (7). Activation of various TLRs stimulates Th1-cell discrimination and inhibits Th2-development (8,9). Therefore, inaccurate TLR expression and role may disrupt the equilibrium of preventive inflammation causing bacterial colonisation or hindered Th1-response/Th2-shift, as noted in AD (10).

Thirteen various human TLRs have been defined to date (5). TLR2 plays a crucial role in connecting microbial products and apoptosis and the host defence mechanism (11). In cooperation with TLR1 and TLR6, TLR2 interplays with cell wall sections of gram-positive bacteria (7); for this reason, TLR2 is a key element in combating gram-positive bacteria (12). *Staphylococcus aureus* was colonised in more than 90% of AD patients (13). TLR2 identifies *S. aureus* and is in charge of host defense against *S. aureus* infection (14). In this respect, impairment in TLR2 signaling has been proposed to play a role in the pathogenesis of AD (15).

Prior studies have reported associations between polymorphisms of the TLR family or connected signal transduction molecules, as well as TLR2 (5,6,7,16,17,18), TLR4 (19), TLR9 (20), toll-interacting protein (21), and AD, although most of those results have not been replicated (22). As a result of the single nucleotide polymorphisms (SNPs) in TLRs or TLR signalling molecules, impairment in the functional responsiveness of TLRs in AD patients may contribute to the pathophysiology of the disease and increased vulnerability of the lesions to bacterial and viral super-infection (23).

Our grasp of the relationship between TLRs and the pathogenesis of disease continues to expand with the further identification of genetic polymorphisms in TLRs and improvement in knowledge of TLR signalling. Using a nominee gene attempt, we investigated whether SNPs in the TLR2 gene c.2258C>T (R753Q) (rs5743708) and TLR2 c.-148+1614T>A (A-16934T) (rs4696480) (NM_0032643) were related to the phenotype of AD in Turkish children.

## MATERIALS AND METHODS

### Study subjects

The study was conducted on 70 Turkish children with AD aged 0.5-18 years, monitored by the outpatient clinic of the hospital’s paediatric allergy department between March 2013 and March 2014. Following the study recommended by Salpietro et al. (7), giving an α level of 0.05 and a power level of 80%, the sample size required was calculated. The inclusion criterion was the presence of AD, diagnosed according to validated criteria (24). The clinical severity of AD was evaluated by the severity scoring of atopic dermatitis (SCORAD) index. AD was mild if the SCORAD index was lower than twenty-five, moderate if between twenty-five and fifty, and severe if higher than fifty (25). Another selection criterion was a steroid-free period of at least one month. None of the patients used systemic or topical antibiotic therapy before examination and none had acute bacterial super-infection. The controls were 69 non-allergic, unrelated outpatients with no individual or family story of allergic illness (asthma, AD, allergic rhinitis, or allergic rhinoconjunctivitis). Specific IgE tests for inhalant or food allergens were negative in the control group.

### Laboratory tests

Serum total IgE levels were measured by chemiluminescent immunometric assay, (Immulite 2000 Allergy; Diagnostic Products Corp., Los Angeles, USA) in the AD and control groups.

Specific IgE antibodies to eight different inhalant allergens (*dermatophagoides pteronyssinus*, dog dander, cat dander, cultivated rye grass, timothy grass, *cladosporium herbarum*, birch, mugwort) were detected qualitatively by chemiluminescent enzyme-labelled immunoassay using Immulite Allergy Inhalant Panel IP8 kit (Diagnostic Products Corp., Los Angeles, USA).

Specific IgE antibodies in a panel of food allergens (milk, egg white, codfish, peanuts, wheat, soybean) were detected qualitatively by chemiluminescent enzyme-labelled immunoassay using Immulite Allergy Food Panel FP5 kit (Diagnostic Products Corporation, Los Angeles, USA). The cut-off value for inhalant and food serum specific IgE levels was above 0.35 kU/L.

### Skin tests

A skin prick test was done in cases with AD using a standard inhalant allergen panel (dust mites, cat fur, dog hair, cockroaches, grass, weed, tree and mould mix) (Alyostal Stallergenes SA, Antony, France) and for food allergens milk, egg white, egg yolk, wheat, hazelnuts, peanuts, walnuts, cacao, fish mixture 1 (sea bream, anchovy, red mullet, sardine), and fish mixture 2 (cod, sole, sea bass, hake) (ALK Abello, Høsholm, Denmark). Atopy was outlined by the presence of the following criteria: a positive skin prick test (wheal width ≥3 mm) from one or more food and/or inhalant allergens or elevated allergen-specific serum IgE (cut-off class ≥1, corresponding to ≥0.35 kU/L).

### Genotyping

Peripheral blood samples of both controls and patients were placed into 2 mL EDTA tubes. Deoxyribonucleic acid isolation from peripheral blood samples was implemented by using Qiagen deoxyribonucleic acid isolation kits (EZ1^®^ DNA Blood 200 µL Kit, Qiagen, Hilden, Germany) with the EZ1 Advanced XL (Qiagen, Hilden, Germany) Nucleic Acid Isolation system. Subsequently, the deoxyribonucleic acid concentration and purity of the isolated deoxyribonucleic acid samples were measured by a NanoDrop device (NanoDrop 2000C, Thermo Scientific, USA). After measuring concentration and purity, amplification PCR for pyrosequencing was done by a PyroMark PCR kit (Qiagen, Hilden, Germany) and primers in the PyroMark Custom Assay kit for the detection of each polymorphism, rs5743708 and rs4696480.

Amplification PCR was performed with an initial denaturation at 95 °C for a quarter-hour, followed by forty-five cycles at 94 °C for thirty seconds, 60 °C for thirty seconds (annealing), and 72 °C for thirty seconds, with a final extension at 72 °C for ten minutes [PyroMark PCR kit (Qiagen, Hilden, Germany) PCR protocol].

After PCR amplification, PCR products were pyro-sequenced with sequencing primers in a PyroMark Custom Assay kit for detection of each polymorphism according to the manufacturer’s instructions (PyroMark Q24 System, Qiagen, Hilden, Germany). Results were then analysed by the PyroMark Q24 software system, and genotypes of samples of both controls and patients were tested for polymorphisms.

### Ethical approval

The study was approved by the native Ethics Committee of our hospital. Written informed consent was obtained from parents and from patients more than seven years of age in the AD and control groups.

### Statistical analysis

Data are given as mean values ± SD or median (minimum-maximum) or number (percentage). Categorical data of the AD and control groups were compared using the Chi-Square test (χ2). Numerical data were compared using the Mann-Whitney U-test. Allele and genotype frequencies in patients and in controls were compared using the χ2 test. P values were estimated to be significant at a level of <0.05. Allele frequencies were tested by Hardy-Weinberg equilibrium.

## RESULTS

### Demographics events

A total of 139 Turkish children were investigated: of those, 70 were AD patients and 69 were healthy controls. Demographic characteristics of the patients are shown in Table 1.

The mean age of the AD group was 3.1±3.8 years, while the mean age of the control group was 6.8±5.9 years. There was a difference between the groups regarding age (p<0.001).

In the AD group, 31 (44.3%) of the cases were female and 39 (55.7%) were male. In the control group, 36 (52.2%) of the patients were female and 33 (47.8%) were male. There was no difference between the groups with regard to gender (p=0.352).

The SCORAD index was 29.9±10.3 in the AD group. According to this index, 24 (34.3%) of the patients had mild, 43 (61.4%) had moderate, and three (4.3%) had severe eczema.

### Serum total IgE and specific IgE levels

Total IgE levels were 245.3±502.2 IU/mL in the AD group and 23.6±18.8 IU/mL in the control group. Serum total IgE levels were significantly higher in the AD group (p<0.001).

In the AD group, specific IgE levels for IPS8 were 5.7±20.0 kU/L, and in the control group were 0.1±0.02 kU/L. Specific IgE levels for inhalant allergens were higher in the AD group than the control group (p<0.001) (Table 1). In the AD group, specific IgE levels for IPS8 were positive in 15 patients. Specific IgE levels for IPS8 were not found to be positive in any of the cases in the control group.

Specific IgE levels for FP5 were 1.0±2.7 kU/L in the AD group and in the control group were 0.1±0.01 kU/L. Specific IgE levels for food allergens were higher in the AD group (p<0.001) (Table 1). In the AD group, specific IgE levels for FP5 were positive in 19 patients. Specific IgE levels for FP5 were not found to be positive in any of the patients in the control group.

### Skin tests

Skin tests were found to be positive for inhalant allergens in 10 patients, for food allergens in nine patients, and for both inhalant and food allergens in cases with AD in nine patients.

### Genetic variation analysis of the patients and controls

We genotyped two different SNPs in the TLR2 gene (R753Q and A-16934T). The results of the genotype analyses are shown in Table 2.

Cytosine-cytosine (CC) and cytosine-thymine (CT) genotype frequencies of the TLR2 R753Q SNP in the AD group were determined as 98.6% and 1.4%, respectively; CC and CT genotype frequencies of TLR2 R753Q SNP in the control group were 95.7% and 4.3%, respectively. In terms of genotype frequency, the groups did not differ from each other (p=0.366) (Table 2).

In the AD group, C allele frequency of the TLR2 R753Q SNP was 99.29% and T allele frequency was 0.71%; in the control group, C and T allele frequencies for the TLR2 R753Q SNP were 97.83% and 2.17%, respectively. There was no difference among the two groups with regard to allele frequency (p=0.853) (Table 2).

In the AD group, thymine-thymine (TT), thymine-adenine (TA), and adenine-adenine (AA) genotype frequencies of the TLR2 A-16934T SNP were 24.3%, 44.3%, and 31.4%, respectively; TT, TA, and AA genotype frequencies of the TLR2 A-16934T SNP in the control group were 18.9%, 47.9%, and 33.2%, respectively. There was no difference between the two groups in terms of genotype frequency (p=0.737) (Table 2).

The T allele frequency of the TLR2 A-16934T SNP in the AD group was 46.43% and the A allele frequency was 53.57%; in the control group, the T and A allele frequencies of the TLR2 A-16934T SNP were 42.75% and 57.25%, respectively. There were no differences between the two groups in allele frequencies (p=0.848) (Table 2).

The TLR2 allele and genotype frequencies were within Hardy-Weinberg equilibrium in AD patients and in control subjects. No homozygous carriers of the TLR2 R753Q SNP were found in the AD and control groups.

## DISCUSSION

TLR2 polymorphisms have been investigated in the field of atopy and allergic diseases (5,6,7,16,17,18,26,27,28,29,30), involving AD (5,6,7,16,17,18,28,30), but the results of published association studies on TLR2 SNPs and the risk of AD are contradictory.

The present study analysed the prevalence of TLR2 genetic variants in a group of Turkish children affected by AD, living in the north-west region of Turkey. The study group’s mean age was 3.1±3.8 years, while it was 6.8±5.9 years for the control group. There was a difference between the groups according to age (p<0.001). Studying the genotype of the cases, we considered that the difference in age would not affect results. We genotyped two different SNPs in the TLR2 gene (R753Q and A-16934T). The TLR2 gene polymorphisms were identically allocated among AD patients and controls, demonstrating that predisposition to AD is not closely related to the genotypes studied here (Table 2).

Salpietro et al. (7) examined TLR2 (R753Q and A-16934T) and TLR4 (D299G and T399I) SNPs in 187 children with AD and 150 healthy children. The frequency of the TLR2 R753Q SNP was higher in AD children compared with controls. The TLR2 R753Q polymorphism was prevalent in patients with severe AD compared with mild-moderate cases. No homozygous carriers of the TLR2 R753Q SNP were found in the AD or the control group. For TLR2 A-16934T polymorphism, the frequency of the homozygous A/A genotype was higher in AD patients compared with controls; however, the TLR2 A-16934 allele was not found to be related to AD severity. Ahmad-Nejad et al. (17) genotyped TLR2 R753Q, TLR2 R677W, TLR4 D299G, and TLR4 T399I SNPs in 78 adult patients with AD and 39 control subjects. They did not find any difference between the frequencies of TLR2 R753Q, TLR2 R677W, TLR4 D299G, and TLR4 T399I SNPs in their AD group and their controls. The frequency of cases carrying TLR2 R753Q or TLR4 SNPs was higher in the AD group compared with the control group (23.5% vs. 5%). Additionally, patients carrying the TLR2 R753Q SNP demonstrated a phenotype characterised by moderate-severe AD.

Oh et al. (16) examined nine SNP frequencies in the genes encoding TLR1, TLR2 (R753Q and A-16934T), TLR4, TLR9, and toll-IL-1 receptor domain-containing adaptor protein in a case/control cohort of 136 adult AD cases and 129 healthy individuals. For all SNPs studied, allelic distribution was analogous among cases and controls.

Potaczek et al. (18) genotyped the TLR2-16934A>T polymorphism in 130 adult AD patients. The SCORAD values of the patients with the 16934TT genotype/-16934T allele were lower than for TLR2-16934AA homozygotes. In individuals with a total serum IgE level ≥106 IU/mL, the relation of the TLR2-16934A>T polymorphism with SCORAD was remarkable. They concluded that the TLR2-16934A>T polymorphism might be a genetic prognosticator of AD severity.

Weidinger et al. (28) studied four TLR2 and eight TLR4 SNPs in a cohort of 275 German, white-parent offspring trios. Total IgE and specific IgE antibodies to common environmental allergens were measured. None of the SNPs showed associations with AD or severity of AD. They could not repeat the reported relationship of AD severity and the TLR2 R753Q polymorphism.

In an Italian study of 159 children (102 eczema and 57 food allergy patients), no evidence of a correlation between the TLR2 R753Q polymorphism and eczema and food allergy incidence and/or severity was found (30).

The present study did not find an association between the TLR2 (R753Q and A-16934T) SNPs and AD. We did not perform further statistical analysis to evaluate the relationship between the severity of AD, total IgE levels, or specific IgE levels and TLR2 (R753Q and A-16934T) SNPs because the polymorphisms were distributed evenly between patients and controls. None of the patients were homozygous for the TLR2 R753Q polymorphism in either the AD or the control group.

Different studies have shown varied results, which could be due to the considerable difference in the frequencies of genetic and non-genetic risk factors within differing populations. It is not possible to generalize conclusions across populations. However, the interaction between genes and environmental factors appear to be significant in allergy development.

Despite the numerous SNPs defined and the lack of data of the functional results of most of them, our attitude was to study supposedly functional SNPs in TLR2 that have been previously studied extensively. Consequently, a possible association between AD and other SNPs in those TLR genes not comprised in this study cannot be excluded. There could also be interplays with other genes not studied here.

The potential role of TLR2 regulating immune responses has been the subject of significant recent attention, although whether these polymorphisms have a direct functional effect on AD pathogenesis is not clear. Further studies involving different cohorts should be carried out to examine the molecular effects of TLR2 SNPs and the association these SNPs may have with AD.

In conclusion, we find lack of association between TLR2 (R753Q and A-16934T) SNPs and AD. Possible associations between AD and other SNPs in TLR genes, as well gene-environment interactions, might play a role in AD aetiology in our patients. Future larger series with other studies conducted by TLR gene sequencing could support our conclusions on this issue.

## Figures and Tables

**Table 1:**
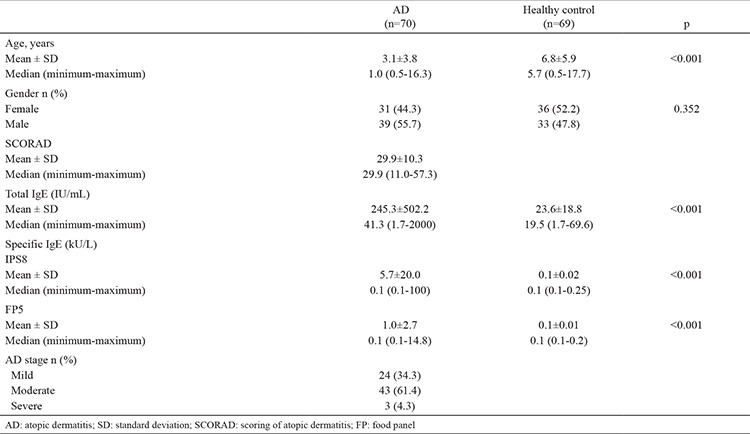
Demographic characteristics of atopic dermatitis patients and healthy control subjects

**Table 2:**
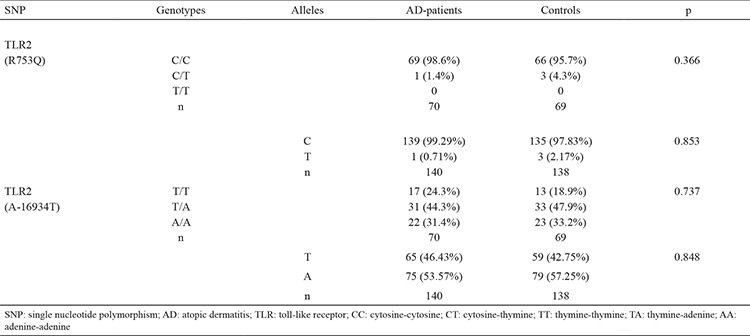
Genotype and allele frequencies of TLR2 (R753Q) and TLR2 (A-16934T) SNPs in AD patients and controls
